# Developing pineapples with an extended shelf life through deletion of the abscisic-acid-responsive element in the enhancer sequence of the 1-aminocyclopropane-1-carboxylate synthase gene AcoACS1

**DOI:** 10.3389/fpls.2026.1769495

**Published:** 2026-02-20

**Authors:** Haiyan Shu, Aiping Luan, You Wang, Junhu He, Qing Wei, Rulin Zhan, Shenghe Chang

**Affiliations:** 1 Tropical Crops Genetic Resources Institute, Chinese Academy of Tropical Agricultural Sciences, Haikou, China; 2 Sanya Research Institute, Chinese Academy of Tropical Agricultural Sciences, Sanya, China; 3 State Key Laboratory of Tropical Crop Breeding, Sanya, China; 4 Key Laboratory of Crop Gene Resources and Germplasm Enhancement in Southern China, Ministry of Agriculture and Rural Affairs, Haikou, China; 5 Key Laboratory of Tropical Crops Germplasm Resources Genetic Improvement and Innovation of Hainan Province, Haikou, China

**Keywords:** 1-aminocyclopropane-1-carboxylic acid synthase gene AcoACS1, abscisic-acid-responsive element, aroma substance, fruit firmness, hydrolase activity

## Abstract

**Introduction:**

Pineapple fruits are frequently discarded because of softening. Non-climacteric fruit ripening is regulated by abscisic acid. Pineapple is a non-climacteric fruit. Ethylene participates in pineapple ripening. However, whether abscisic acid regulates pineapple ripening and the relationship between abscisic acid and ethylene in pineapple fruit have not been reported.

**Methods:**

Pineapple mutant lines were generated using the clustered regularly interspaced short palindromic repeats (CRISPR)/CRISPR-associated protein 9 (Cas9). The ethylene content, abscisic acid content, respiration rate, fruit firmness, aroma profiles, and the activities of polygalacturonase, pectin methylesterase, β-galactosidase, xylanase, and cellulase in pineapple flesh were measured. Quantitative real-time reverse transcription polymerase chain reaction was performed to analyze the expression of the 1-aminocyclopropane-1-carboxylate synthase gene AcoACS1 across different pineapple lines.

**Results and discussion:**

Both abscisic acid and ethylene can induce pineapple ripening and softening. Ethylene content in pineapple fruits can be stimulated by abscisic acid. An abscisic-acid-responsive element in the enhancer sequence of the 1-aminocyclopropane-1-carboxylic acid synthase gene AcoACS1 was identified. After this element was knocked out, the expression of AcoACS1 was not induced by abscisic acid treatment, and the onset of pineapple softening was delayed. Aroma compound contents in the mutant fruit and Tainong 17 were similar. Abscisic acid induces pineapple ripening via the abscisic-acid-responsive element in AcoACS1. Pineapples with a long shelf-life can be generated through knocking out this element in AcoACS1.

## 1 Introduction

Pineapple is the most traded tropical fruit in terms of global volume (Xie et al., 2018; Wang et al., 2017). In China, the pineapple harvest season often overlaps with those of citrus and litchi, resulting in market saturation and causing many pineapples to remain unsold. Pineapple fruits harvested at 80% maturity can only be stored at 25°C for 7 to 10 days. Approximately 20% of pineapples in China are discarded each year due to softening and decay. Thus, investigating strategies to extend the shelf life of pineapples is of great importance.

Ethylene and abscisic acid (ABA) play important roles in fruit softening. The softening of climacteric fruits is primarily regulated by ethylene (Chen et al., 2018), while the softening of non-climacteric fruits is mainly regulated by ABA (Fan et al., 2022). However, this conclusion is derived primarily from studies on a limited number of species. It remains unclear whether it applies to other non-climacteric fruits. In strawberry, for instance, ethylene treatment can delay anthocyanin accumulation at the green stage (Figueroa et al., 2021), while ripening at the white stage can be inhibited by 1-methylcyclopropene (1-MCP), an ethylene inhibitor (Villarreal et al., 2009). Furthermore, ABA treatment has been shown to induce ethylene production in strawberry (Jiang and Joyce, 2003). Collectively, these findings demonstrate that ethylene also participates in the ripening process of strawberry. Similar phenomena have been observed in grape and orange as well (Wang et al., 2022; Sun et al., 2025).

Pineapple softening has a close relationship with ABA and ethylene. When pineapple fruits were harvested at 80% maturity and sprayed with 200 mg/L of ABA, the occurrence of internal browning could be delayed (Zhang et al., 2016b, Zhang et al., 2015). Internal browning of pineapples is closely related to fruit softening (Hong et al., 2017). After pineapple fruits at 80% maturity were sprayed with 0.45 μL/L of 1-MCP, the shelf life of pineapples was extended by 3–5 days (Zhang et al., 2016a). Ethylene may play a role in pineapple softening.

ABA promotes the degradation of cell walls, leading to fruit softening (Han et al., 2016). At the same time, postharvest treatment of pineapple with ABA is necessary to delay the occurrence of internal browning (Zhang et al., 2016b, Zhang et al., 2015). This presents a dilemma regarding whether to treat pineapple fruits with ABA. Additionally, ABA biosynthesis in plants involves more steps and genes than that of ethylene (Dong et al., 2015; Larsen, 2015). Developing pineapples with an extended shelf life by regulating ethylene content in fruit may be a more feasible strategy.

Ethylene synthesis in plants involves multiple steps. Initially, methionine is converted to S-adenosylmethionine, which is subsequently transformed into 1-aminocyclopropane-1-carboxylic acid (ACC) and methylthioadenosine catalyzed by ACC synthase. ACC is then oxidized to ethylene in the presence of oxygen and ACC oxidase (Larsen, 2015; Park et al., 2018). ACC synthase is the key enzyme in ethylene biosynthesis in plants (Karagiannis et al., 2018; Sadka et al., 2019). The expression of the tomato ACC synthase gene ACC-S was suppressed, and the ACCS-antisense-suppressed tomato line was constructed (Sozzi et al., 2001). At 78 days after flowering, the control fruits began to soften and rot, while the fruits of the ACCS-antisense-suppressed line remained firm (Sozzi et al., 2001).

The pineapple genome contains two ACC synthase genes. Through gene expression co-suppression, a partial fragment of the pineapple ACC synthase gene AcoACS2 was introduced into the ‘Spineless Cayenne’ cultivar (Trusov and Botella, 2006), generating two AcoACS2-silenced lines. Under field conditions, the natural flowering rates of these silenced lines were similar to those of the control; however, the onset of flowering was significantly delayed compared to the control (Trusov et al., 2006). While low levels of AcoACS1 expression are detected in green fruits, a 16-fold increase occurs in ripe fruit tissue (Cazzonelli et al., 1998). This upregulation of AcoACS1 is likely the primary cause of elevated ethylene content in yellow pineapple fruits, potentially serving as the trigger for softening. In this research, an ABA-responsive element (ABRE) in the enhancer sequence of AcoACS1 was identified. After this element was knocked out, the expression of AcoACS1 was not induced by ABA treatment, and the onset of pineapple softening was delayed. Aroma compound contents in the ABRE-knockout mutant fruit and Tainong 17 were similar. ABA induces pineapple ripening via the ABRE in AcoACS1. To our knowledge, this is the first paper to report generating a long shelf-life non-climacteric fruit by mutating the ACC synthase gene, and it is also the first paper to report that ABA promotes non-climacteric fruit softening via stimulation of ethylene synthesis. These provide new insights into the mechanisms of ripening and softening in non-climacteric fruits.

## 2 Materials and methods

### 2.1 Materials

“Tainong 17” pineapple was used in this research, which was planted in the Danzhou experiment field of Tropical Crops Genetic Resources Institute, Chinese Academy of Tropical Agricultural Sciences. All chemical reagents were purchased from the Sigma-Aldrich Company (Shanghai, China).

### 2.2 Generation of pineapple AcoACS1 mutants

Plasmids pYLsgRNA-OsU3m (Plasmid#66193) and pYLCRISPR/Cas9Pubi-H (Plasmid#66187) were purchased from Addgene (USA). The transformation vector was constructed according to a published protocol (Ma et al., 2015). According to the genomic DNA sequence of AcoACS1 (GenBank accession number NC_033623.1), 5′-gaggattcgccttactttga-3′ was selected as the target sequence. Oligo A (5′-CTTGaggattcgccttactttgaGTTTTAGAGCTAGAAATAGC-3′) and oligo B (5′-AAACtcaaagtaaggcgaatcctCAAGC-3′) were synthesized. For annealing, 1 μL of Oligo A (100 μM), 1 μL of Oligo B (100 μM), 1 μL of 10× T4 ligase buffer, and 7 μL of nuclease-free water were mixed. The mixture was incubated at 95°C for 5 min, then slowly cooled to 25°C (at a rate of 0.1°C/min), and finally held at 4°C. The annealed double-stranded oligonucleotide was inserted into pYLsgRNA-OsU3m. The reaction mixture contained 50–100 ng of pYLsgRNA-OsU3m plasmid, 1 μL of the diluted annealed oligonucleotide (diluted 10-fold before use), 1 μL of *Bsa*I-HFv2 restriction enzyme, 1 μL of T4 DNA ligase, and 2 μL of 10× T4 DNA ligase buffer, made up to a final volume of 20 μL with sterile water. The reaction conditions were as follows: 20 cycles of 37°C for 5 min and 16°C for 10 min, followed by 37°C for 30 min and 80°C for 10 min. The reaction product was transformed into competent *Escherichia coli* (DH5α) cells, which were then spread on LB plates containing 100 µg/mL of spectinomycin. Single colonies were picked and cultured, and plasmids were extracted. Sequencing was performed using the OsU3 promoter universal primer. The verified plasmid was designated as pYLsgRNA-A.

Subsequently, a second round of Golden Gate Assembly (a one-step cloning technique that utilizes type IIS restriction endonucleases and DNA ligase to directionally assemble multiple DNA fragments efficiently and seamlessly within a single reaction system) was performed to assemble the sgRNA expression cassette into the destination vector. The reaction mixture contained 100 ng of pYLCRISPR/Cas9Pubi-H plasmid, 50 ng of pYLsgRNA-A plasmid, 1 μL of *Bsa*I-HFv2 restriction enzyme, 1 μL of T4 DNA ligase, 2 μL of 10× T4 DNA ligase buffer, and sterile water added to a final volume of 20 μL. The reaction conditions were as follows: 20 cycles of 37°C for 5 min and 16°C for 10 min, followed by 37°C for 30 min and 80°C for 10 min. This step excised the entire sgRNA (single-guide RNA) expression cassette from pYLsgRNA-A and ligated it into pYLCRISPR/Cas9Pubi-H. The product was transformed into *E. coli* (DH5α). The transformed *E. coli* mixture was spread on LB plates containing 75 µg/mL of kanamycin. Single colonies were picked, and plasmids were extracted. PCR was performed using primers P1 (5′-TGCCTGATCCCTTCAGGAG-3′) and P2 (5′-GCGGTAGCCAATTATACCTT-3′) to confirm correct assembly. The verified plasmid was named pYLCRISPR/Cas9Pubi-H-A. This plasmid was introduced into *Agrobacterium tumefaciens*-competent cells (EHA105) via electroporation. Embryogenic callus was induced and transformed following the protocol in a published paper (Shu et al., 2024). Kanamycin (20 mg/L) was used to select transformed callus. To confirm the transformation of pineapple plants, PCR was performed using primers Cas9-F (5′-GAGGGCAAGAATGGGAATAG-3′) and Cas9-R (5′-GTGGCTCGACGTCTACTTCA-3′) with genomic DNA extracted from putative transgenic plants as templates. Successful transformation was indicated by the presence of a 650-bp band on agarose gel. To identify mutations in the target sequence, PCR was performed using primers P3 (5′-tggtgattgctgatggggcg-3′) and P4 (5′-cggcaagccatggtagtctt-3′) with genomic DNA extracted from the transgenic pineapple plants as templates. The PCR products were sent to Sangon Biotech (Shanghai, China) for sequencing.

### 2.3 Generation of pineapple AcoACS1-ABRE knockout mutants

Plasmids pYLsgRNA-OsU3m (Plasmid#66193) and pYLCRISPR/Cas9Pubi-H (Plasmid#66187) were purchased from Addgene (USA). The vector for transformation was constructed according to a published protocol (Ma et al., 2015). According to the genomic DNA sequence of AcoACS1 (NC_033623.1), 5′-cgctggagttaatacgtgag-3′ was selected as the target sequence. Oligo A (5′-CTTGgctggagttaatacgtgagGTTTTAGAGCTAGAAATAGC-3′) and oligo B (5′-AAACctcacgtattaactccagcCAAGC-3′) were synthesized. The annealing of double-stranded oligonucleotides, cloning into pYLsgRNA-OsU3m, and subcloning into pYLCRISPR/Cas9Pubi-H were performed as described in Section 2.2. The transformed *E. coli* mixture was spread on LB plates containing 75 µg/mL of kanamycin. Single colonies were picked, and plasmids were extracted. PCR was performed using primers P1 (5′-TGCCTGATCCCTTCAGGAG-3′) and P2 (5′-GCGGTAGCCAATTATACCTT-3′). The PCR products were sent to Sangon Biotech (Shanghai, China) for sequencing. The verified plasmid was named pYLCRISPR/Cas9Pubi-H-ABRE. Subsequent steps were performed, including the transformation of pYLCRISPR/Cas9Pubi-H-ABRE into *A. tumefaciens* (EHA105), embryogenic callus induction, infection, and the identification of transgenic plants and AcoACS1-ABRE-knockout mutants, as described in Section 2.2.

### 2.4 Determination of hormone content

Ethylene production in pineapple fruits was measured according to the protocol described previously (Zeng et al., 2020). A GC2010 gas chromatograph (Shimadzu, Kyoto, Japan) equipped with a flame ionization detector was used. Measurements were performed in triplicate for each sample, with each sample consisting of five to eight fruits.

### 2.5 Determination of respiration rate

The respiration rate was measured using the static alkaline absorption method (Ye et al., 2014). Two glass desiccators of identical volume were prepared. A Petri dish containing 20 mL of 0.4 mol/L NaOH solution was placed inside each desiccator. A perforated platform was then placed above the dish, and four pineapple fruits (with pre-measured weight and volume) were placed on the platform in one desiccator. The other desiccator served as a blank control and was filled with an equal volume of cooled distilled water. After 1 h of incubation, the Petri dishes were removed, and their contents were transferred into conical flasks. Saturated BaCl_2_ solution and phenolphthalein indicator were added, followed by titration with 0.1 mol/L of standard oxalic acid solution. The blank sample was titrated in the same manner.

### 2.6 Quantitative real-time polymerase chain reaction

Quantitative real-time polymerase chain reaction (qRT-PCR) was performed according to a published protocol (Shu et al., 2024). Pineapple fruits were collected and placed in a room at 25°C with 85% relative humidity. Total RNA was extracted using TRIzol reagent (Invitrogen, USA). RNA was reverse-transcribed into cDNA using the PrimeScript™ RT reagent kit (TaKaRa, Dalian, China). The housekeeping gene AcoTubulin (Aco010170.1) was used as the internal control. Each real-time PCR reaction contained SYBR^®^ Premix Ex Taq II (Tli RNaseH Plus), forward and reverse primers, cDNA template, and ddH_2_O. All PCR reactions were performed in triplicate. The relative mRNA expression levels of each gene were analyzed using the 2^-^^ΔΔCt^ method and normalized to the reference gene AcoTubulin.

### 2.7 Determination of fruit firmness

Fruit firmness was measured according to a published method (Zhang et al., 2022). A TMS-Pro food property analyzer (FTC, USA) was used. Measurements were taken at three positions: the basal end (base), the middle section, and the apical end (far end) of the fruit. The average value of these measurements was calculated. Six fruits were measured for each treatment.

### 2.8 Measurement of aroma substances

Pineapple fruits at 80% maturity were collected. An Agilent 6890–5975 gas chromatography-mass spectrometry (GC-MS) system (Agilent Technologies, USA), a manual SPME sampler (Supelco, USA), and a 65-μm PDMS solid-phase microextraction fiber (Supelco, USA) were used. Pineapple pulp was homogenized, and 6.5 g was placed into a sample vial, which was sealed with a PTFE-lined butyl rubber septum; an empty vial served as a control. Headspace solid-phase microextraction (HS-SPME) was used to extract aroma components for injection. The sample vial was incubated at 50°C for 40 min. After extraction, the fiber was inserted into the GC injection port and desorbed at 250°C for 2.5 min. Chromatographic separation was performed on an HP-5 column (30 m × 0.32 mm × 0.25 μm). The injection port temperature was 200°C, and splitless injection was used. The column temperature program was initiated at 35°C (held for 2 min), increased to 180°C at a rate of 8°C/min (held for 4 min), and finally increased to 230°C at 15°C/min (held for 1 min). The mass spectrometry conditions were as follows: helium was used as the carrier gas at a flow rate of 1.06 mL/min; electron ionization (EI) was employed with an electron energy of 70 eV; the ion source temperature was 230°C; and mass spectra were acquired in full scan mode with a scan range of *m*/*z* 45–450. Compounds were identified by comparing their mass spectra with the NIST05 library and through manual interpretation. The relative content of each component was calculated using the peak area normalization method. Quantification was performed using the internal standard method with n-tetradecane (3.85 μg) added as the internal standard. Data were subjected to statistical analysis using SPSS software, with P <0.05 considered statistically significant.

### 2.9 Polygalacturonase activity measurement

Polygalacturonase (PG) activity was determined according to a published protocol (Ye et al., 2014). Five grams of frozen pineapple pulp was homogenized in 50 mmol/L of sodium phosphate buffer (pH 6.0) containing 2.4 mol/L of NaCl, 0.5 g of PVP, and a small amount of quartz sand. The mixture was ground at 4°C for 2 min and then centrifuged at 10,000 rpm for 30 min at 4°C. The supernatant was transferred to a dialysis bag (12,800 Da MW cutoff cellulose membrane). Dialysis was performed against 50 mmol/L of sodium phosphate buffer (pH 4.5) containing 150 of mmol/L NaCl for 24 h, and the buffer was changed once during the process. The dialyzed supernatant was used as the crude PG enzyme extract. For the reaction, 0.1 mL of crude PG enzyme was added to 0.4 mL of 0.2% pectin solution. The reaction mixture was incubated in a water bath at 40°C for 1 h. Then, 1.5 mL of 3,5-dinitrosalicylic acid (DNS) reagent was added, and the mixture was incubated in a boiling water bath for 10 min. After cooling to room temperature, the mixture was diluted 5-fold. The absorbance was measured at 540 nm. A standard curve was constructed using galacturonic acid as the standard.

### 2.10 Determination of pectin methylesterase activity

Pectin methylesterase (PME) was extracted, and the activity was measured according to a published method (Ye et al., 2014). Twenty grams of frozen pulp was homogenized for 2 min at 4°C with 40.0 mL of a pre-cooled mixture containing 2 mol/L of NaCl, 0.50 g of PVP, and a small amount of quartz sand. The homogenate was centrifuged at 10,000 rpm for 30 min at 4°C. The supernatant was dialyzed against 0.1 mol/L of sodium phosphate buffer (pH 7.5) for 24 h. The dialyzed supernatant was used as the crude PME enzyme extract. For the activity assay, 0.3 mL of 0.5% pectin solution (dissolved in 0.1 mol/L of sodium acetate buffer, pH 5.5) was mixed with 0.1 mL of PME extract and 0.6 mL of 0.1 mol/L sodium acetate buffer (pH 5.5). The reaction mixture was incubated at 30°C for 1 h. After incubation, the solution was cooled to room temperature, and 2 mL of potassium permanganate–phosphoric acid solution was added. The mixture was allowed to stand at room temperature for 90 min. Then, 2 mL of 10% oxalic acid solution was added, and the solution was heated until the purple color disappeared. Finally, 2 mL of acetylacetone solution was added, and the mixture was heated in a boiling water bath for 5 min. After cooling to room temperature under running tap water, the absorbance was measured at 418 nm. A boiled enzyme solution was used as a blank control. A standard curve was constructed using anhydrous methanol.

### 2.11 β-Galactosidase activity assay

β-Galactosidase (β-Gal) activity in pineapple was measured according to a published protocol (Ye et al., 2014). Five grams of frozen pulp was homogenized for 2 min at 4°C with 20.0 mL of 1.4 mol/L NaCl solution (pH 6.0), 0.50 g of PVP, and a small amount of quartz sand. The mixture was stirred for 1 h at 4°C, followed by centrifugation at 10,000 rpm for 30 min at 4°C. The supernatant was dialyzed against 0.1 mol/L of citrate buffer (pH 4.0) for 24 h. The resulting supernatant served as the crude β-Gal enzyme extract. For the activity assay, 0.1 mL of the crude extract was mixed with 0.4 mL of bovine serum albumin (BSA) solution, 0.5 mL of 0.1 mol/L citrate buffer (pH 4.0), and 0.4 mL of 16 mmol/L p-nitrophenyl-β-D-galactopyranoside (PNPG) dissolved in 0.1 mol/L of citrate buffer. The reaction mixture was incubated at 37°C for 5 min. The reaction was terminated immediately by adding 2 mL of 0.2 mol/L Na_2_CO_3_. The absorbance of the released p-nitrophenol was measured at 400 nm. A blank control was prepared by adding 2 mL of 0.2 mol/L Na_2_CO_3_ to the enzyme mixture prior to the addition of the substrate. A standard curve was constructed using p-nitrophenol.

### 2.12 Xylanase activity assay

Xylanase (XYL) activity in pineapple was measured according to a published method (Ye et al., 2014). Twenty-five grams of frozen pulp was homogenized for 2 min at 4°C in 50 mL of 0.05 mol/L sodium acetate buffer (pH 5.0) containing 0.1 mol/L of NaCl, 2% (v/v) dithiothreitol (DTT), and 5% (w/v) PVP. The homogenate was centrifuged at 8,000 rpm for 30 min at 4°C. The supernatant was collected as the crude XYL extract. For the activity assay, 0.5 mL of the crude extract was mixed with 1.3 mL of 0.1 mol/L sodium acetate buffer (pH 5.0) and 0.2 mL of 0.1% (w/v) xylan (prepared in 0.1 mol/L of sodium acetate buffer, pH 5.0). The reaction mixture was incubated at 37°C for 1 h. The released reducing sugars (xylose) were quantified using DNS, with boiled, heat-inactivated crude XYL extract serving as the blank control. A standard curve was constructed using xylose.

### 2.13 Cellulase activity assay

Cellulase (CX) activity in the pulp was measured according to a published method (Ye et al., 2014). Five grams of frozen pulp was homogenized for 2 min at 4°C in 20.0 mL of pre-cooled 0.04 mol/L sodium acetate buffer (pH 4.6) containing 6.8% NaCl and 1.8 mmol/L of EDTA. The mixture was stirred at 4°C for 1 h. Subsequently, the homogenate was centrifuged at 10,000 rpm for 15 min at 4°C, and the supernatant was dialyzed against 0.04 mol/L of sodium acetate buffer (pH 4.6) at 4°C for 24 h. The dialyzed supernatant served as the crude CX extract. For the activity assay, 0.5 mL of the crude extract was incubated with 0.5 mL of 1% carboxymethyl cellulose (CMC) solution at 40°C for 1 h. The reaction was terminated by adding 2.5 mL of DNS reagent, followed by boiling for 5 min. After cooling to room temperature, the absorbance was measured at 540 nm. A standard curve was constructed using glucose.

### 2.14 Prediction of off-target sites

Potential off-target sites were predicted using Cas-OFFinder according to a published method (Bae et al., 2014). The target sequences were entered into the ‘Query Sequences’ box to identify potential off-target loci (http://www.rgenome.net/cas-offinder/). Primers flanking these sites were designed for PCR amplification and sequencing. The resulting sequences were aligned with the pineapple reference genome to identify off-target mutations.

### 2.15 Statistical analysis

Each treatment consisted of three replicates. Significant differences among the treatments were analyzed using analysis of variance (ANOVA) followed by Duncan’s multiple range test (DMRT). Statistical analysis was performed using SPSS software. Differences were considered significant at *P <*0.05. Data are presented as mean ± standard error (SE).

## 3 Results

### 3.1 Generation of pineapple AcoACS1-mutant lines

**Figure 1:**
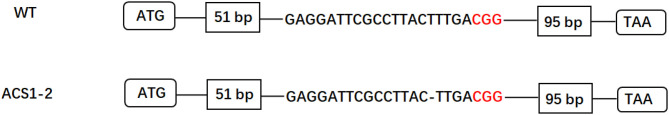
Partial cDNA sequence of the AcoACS1 gene in pineapple lines. The wild-type (WT) control was Tainong 17. The protospacer adjacent motif (PAM) is highlighted in red. The target sequence is shown before the PAM site. Putative premature stop codons are indicated in the last frames. Deleted base is represented by a dash.

**Figure 2:**
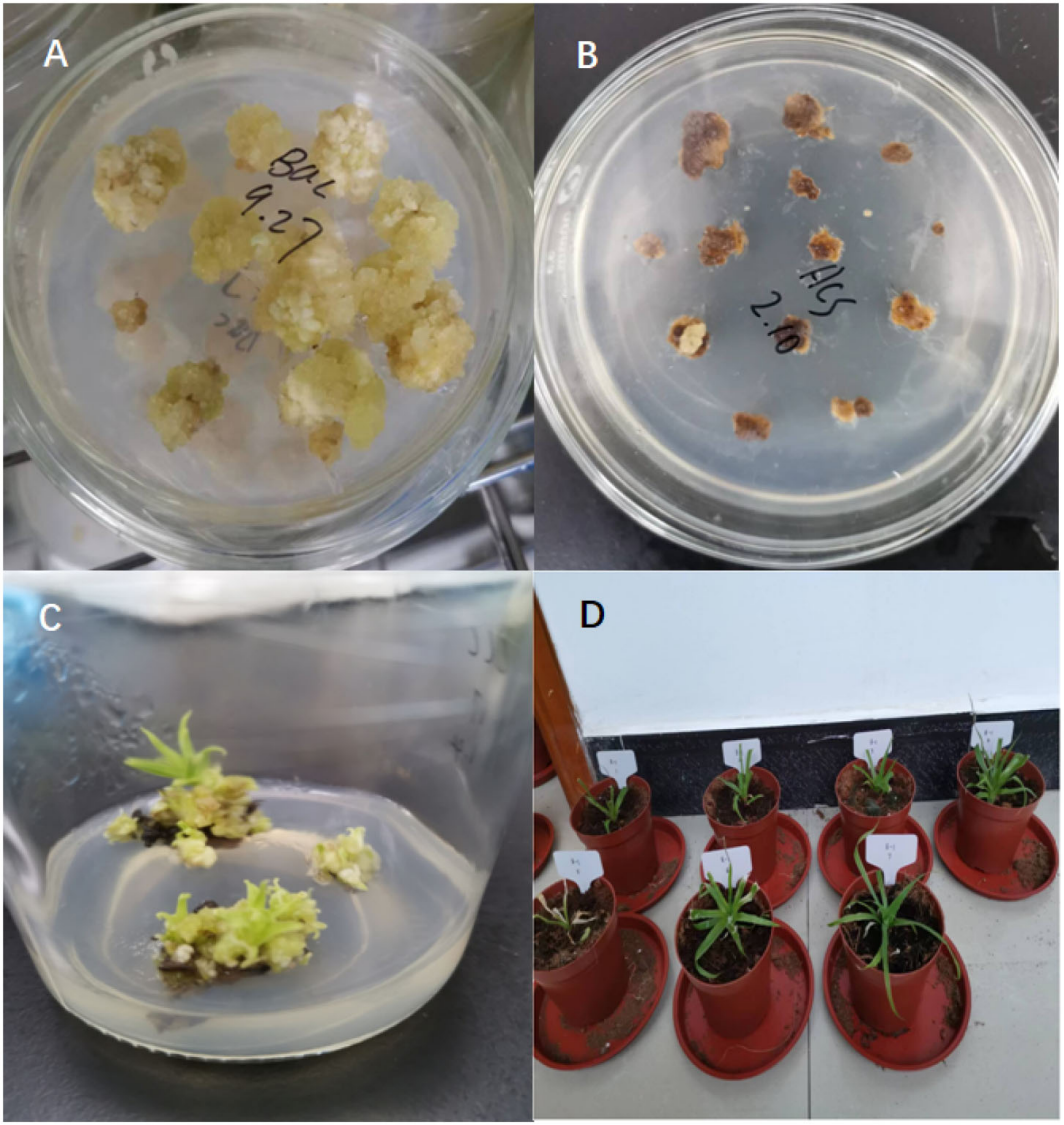
Generation of pineapple AcoACS1 mutants. **(A)** Embryogenic callus of Tainong 17. **(B)** Transformed callus cultured on medium containing 20 mg/L of kanamycin. **(C)** Seedlings differentiated from the transformed callus grown on medium containing 20 mg/L of kanamycin. **(D)** Edited pineapple lines grown in pots.

To investigate whether AcoACS1 is the key gene responsible for pineapple softening, pineapple AcoACS1 mutants were generated using CRISPR/Cas9. The target sequence was selected based on two criteria: first, proximity to the 5′ end to maximize the effects of gene disruption; and second, a low number of predicted off-target sites to facilitate screening. Based on these criteria, the sequence 5′-gaggattcgccttactttga-3′ was selected for sgRNA construction (**Figure 1**). Following the transformation and differentiation of embryogenic calli, 17 transgenic plants were obtained (**Figure 2**). PCR amplification and sequencing of the target regions revealed that five plants were successfully edited. The editing rate was 29%. These lines were designated ACS1–1 through ACS1-5 (**Figures 1**; **Supplementary Figure S1**). Among the five lines, ACS1–2 was found harboring a premature termination codon (**Figure 1**). Off-target analysis was performed using a published method (Bae et al., 2014), and no off-target mutations were detected in the pineapple genome. Consequently, ACS1–2 was selected for further experiments.

### 3.2 ACS1–2 pineapple has a significantly longer shelf life than Tainong 17

**Figure 3:**
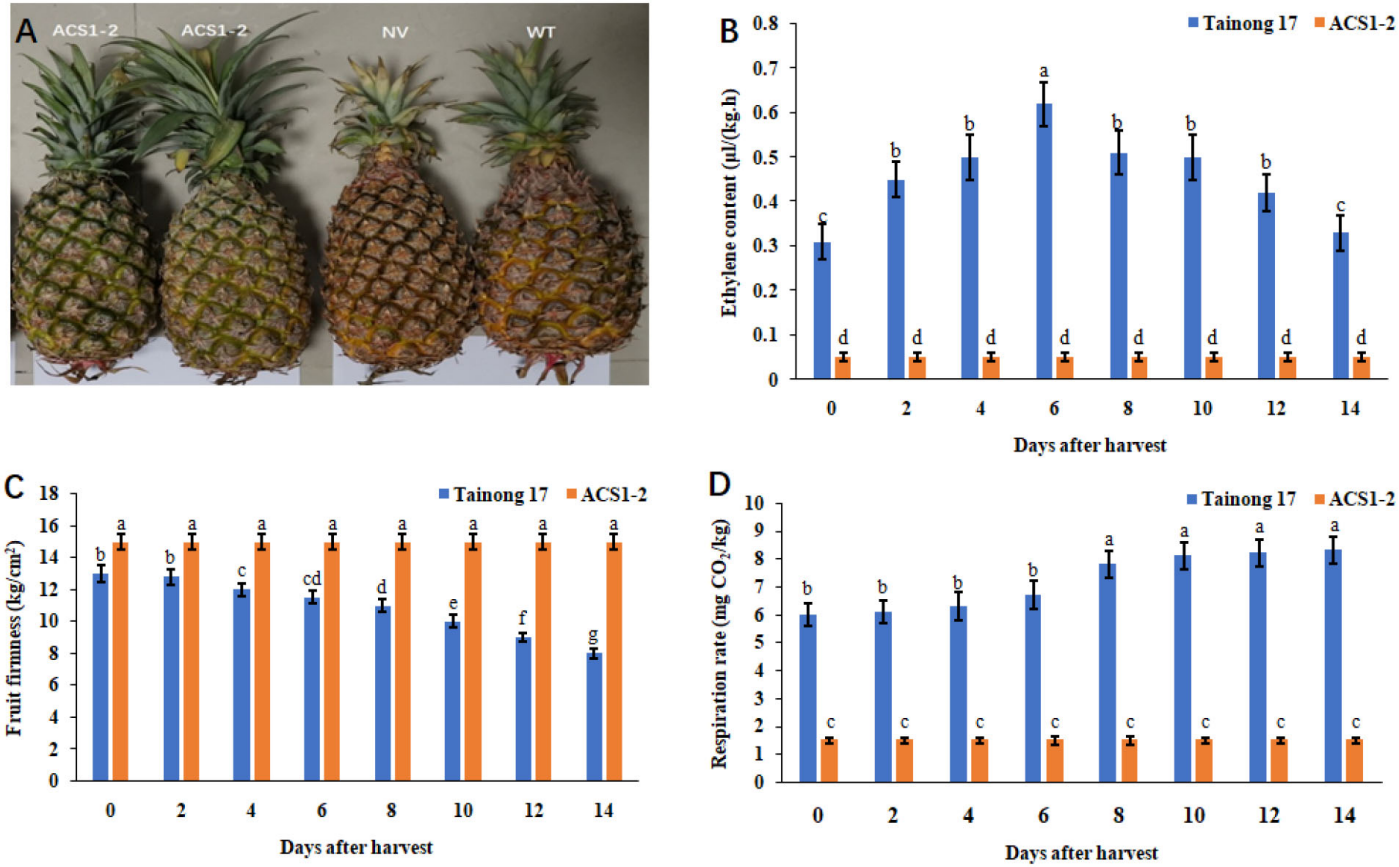
Fruit phenotypes of pineapple lines. **(A)** Fruits stored at 25°C with 85% relative humidity for 30 days. NV represents pineapple fruit transformed with the empty vector. WT represents Tainong 17. **(B)** Ethylene content in pineapple fruits. **(C)** Fruit firmness of pineapples. **(D)** Respiration rate of pineapple fruits. Pineapple fruits were harvested at the 80% maturity stage and stored in a room at 25°C with 85% relative humidity. The measurements were performed with three replicates for each treatment, each consisting of five to eight fruits. Data are the means ± SE (standard error). Different letters indicate significant differences, which were analyzed according to Duncan’s test (*P* < 0.05).

**Figure 4:**
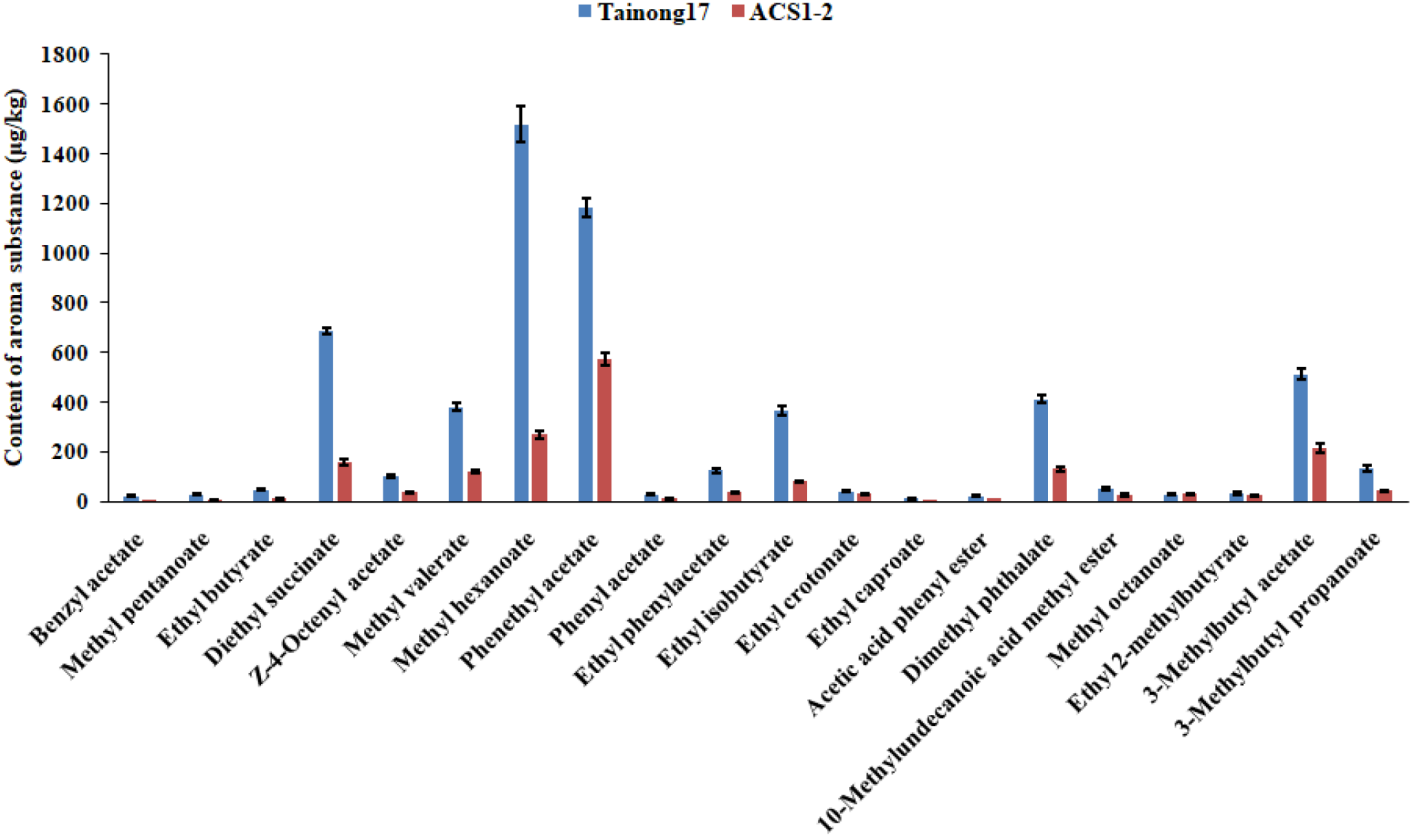
Contents of aroma compounds in pineapple flesh. Pineapple fruits were collected at the 80% maturity stage, and the contents of aroma compounds in pineapple flesh were measured. The measurements were performed with three replicates for each sample, including five to eight fruits per sample. Data are the means ± SE. Significant differences were analyzed according to Duncan’s test (*P* < 0.05).

The ACS1-2 plants exhibited no significant differences in phenotypes compared to Tainong 17. The flowering time and fruit shape were also similar (**Figure 3A**). However, the ethylene content in ACS1–2 fruits was significantly lower than that in Tainong 17 (**Figure 3B**). ACS1–2 fruits remained firmer and greener than Tainong 17, staying in an unripe state for a long time (**Figure 3C**). The respiration rate of the ACS1–2 fruit was remarkably slower than that of Tainong 17 (**Figure 3D**). ACS1–2 has a significantly longer shelf life than Tainong 17. The firmness of the ACS1–2 fruit showed no significant change within 60 days, after which it decreased. However, the contents of many aroma compounds in ACS1–2 fruits were much lower than those in Tainong 17 (**Figure 4**). Its eating quality was less than that of Tainong 17. Before sale, ethephon application was required for ACS1–2 fruits, which increased cost and inconvenience.

### 3.3 ABA-responsive element may determine ethylene content in pineapple fruit

**Figure 5:**
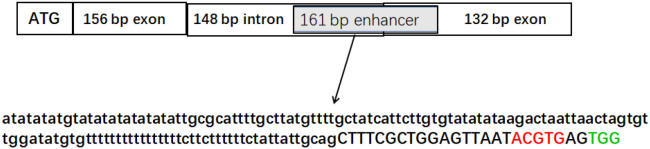
Partial genomic sequence of AcoACS1. The introns were shown in lowercase letters, while the exons were shown in capital letters. The ABRE sequence is shown in red capital letters. The PAM site for constructing the ABRE-knockout line is shown in green capital letters.

In the genomic sequence of AcoACS1, 171 bp from the start codon, there is a 161-bp sequence containing an ABA-responsive element (ACGTG) (ABRE) and an AT-rich region, which are the characteristics of a typical enhancer (**Figure 5**; **Supplementary Figure S2**). This sequence may play an important role in regulating the transcription of AcoACS1 in pineapple fruit, as well as influencing ethylene content. ABREs often appear in the promoters of plant genes, where they mediate ABA responses (Mishra et al., 2014). Therefore, the ABRE may play a key role in regulating AcoACS1 expression. Knocking out the ABRE may reduce enhancer activity and ethylene content in pineapple fruits without destroying the function of AcoACS1. This would allow the fruits to maintain a certain amount of ethylene, achieving the goal of significantly reducing the softening rate without significantly decreasing the contents of aroma compounds or affecting edible quality.

### 3.4 Pineapple ABRE-knockout mutant was generated

**Figure 6:**
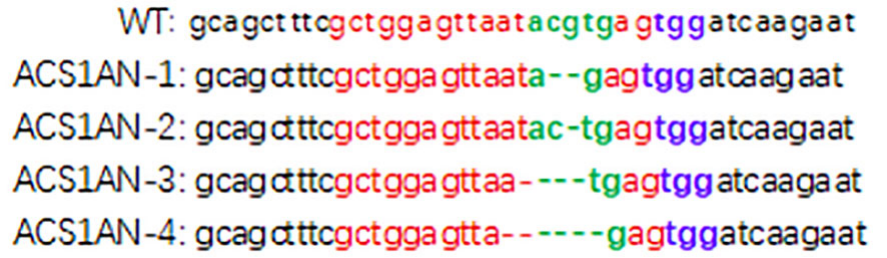
Target sequences in pineapple AcoACS1 ABRE-knockout lines. The ABRE sequence is shown in green letters. The PAM site is shown in blue letters. Deleted bases are indicated by dashes.

**Figure 7:**
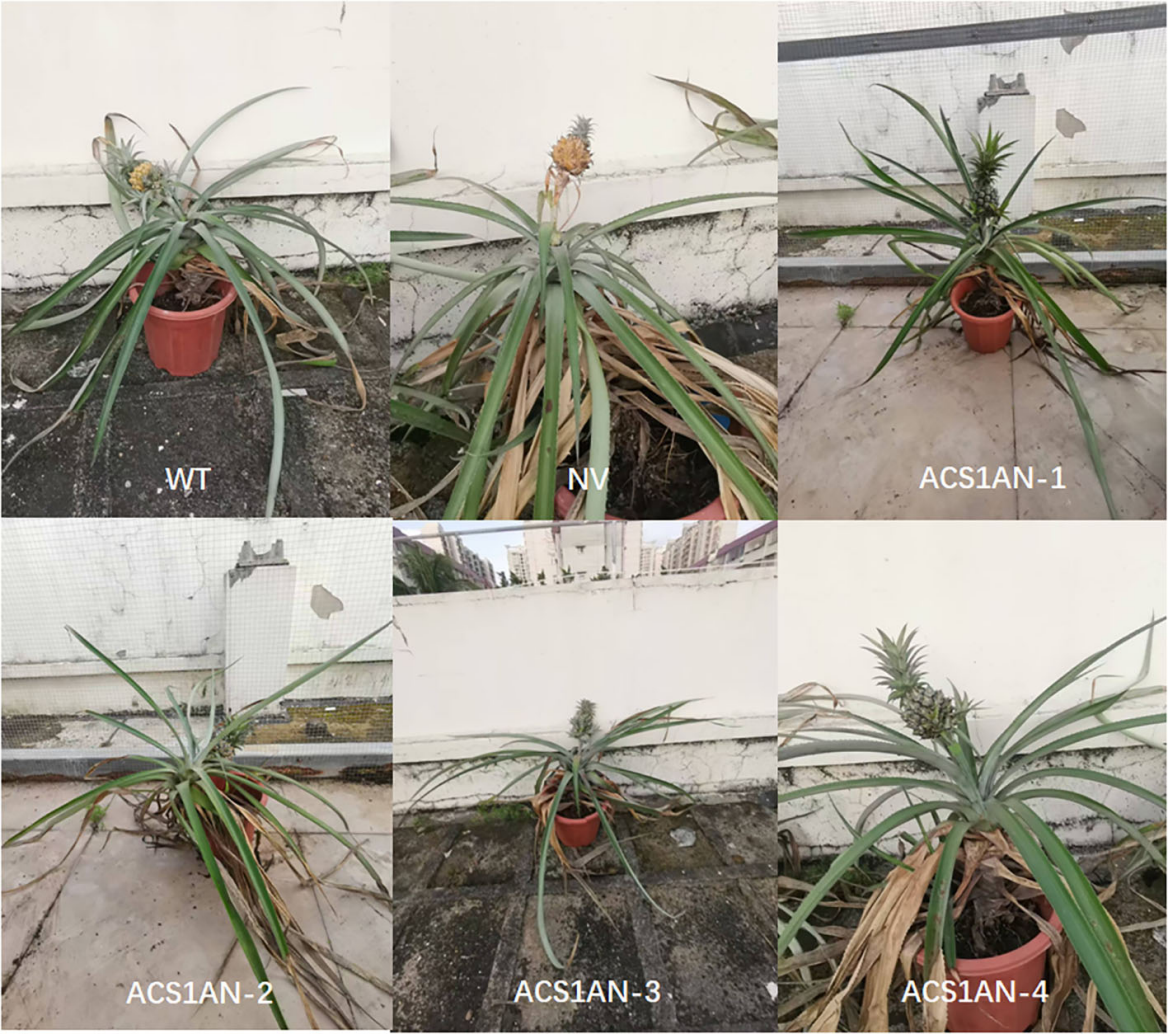
Pineapple AcoACS1-ABRE-knockout lines grown in pots. WT refers to Tainong 17. NV refers to pineapple plants transformed with the empty vector.

To identify whether the ABRE in AcoACS1 determines ethylene content in pineapple fruit, pineapple ABRE-knockout mutants were generated using the CRISPR/Cas9 system. In generating the mutants, the target for deletion was ACGTG. According to the principle for designing sgRNAs, a PAM sequence should be located 3–4 bp downstream of the deletion target. No suitable PAM was identified immediately adjacent to the 3′ end of ACGTG, rendering the direct deletion of this specific sequence infeasible. Although a CGG site was identified 19 bp downstream, the distance between the target sequence and this PAM exceeded the optimal editing window (3–13 bp), suggesting a very low probability of successful deletion. However, by shortening the deletion target to the ACGT motif within the broader sequence 5′-agttaatacgtgagtggatcaag-3′, a TGG PAM site was identified 3 bp downstream of the target. Although the ACGTG ABRE site was not completely removed, the deletion of the ACGT segment is sufficient to disrupt the ABRE function. Therefore, in this study, ACGT within 5′-agttaatacgtgagtggatcaag-3′ was selected as the deletion target. 5′-cgctggagttaatacgtgag-3′ was selected as the guide sequence for constructing the sgRNA. After the embryogenic callus was transformed and differentiated, 21 transgenic pineapple plants were obtained. Target sequences were amplified and sequenced, revealing that four plants had been edited successfully. They were named ACS1AN-1 to ACS1AN-4. The editing rate was 19%. Among the four plants, the ACGT segment in the ABRE sequence in ACS1AN-4 was successfully knocked out (**Figures 6**, **7**). Based on the method published for predicting off-target sites (Bae et al., 2014), no possible off-target sequence was found in the pineapple genome. ACS1AN-4 was chosen for subsequent experiments.

### 3.5 The transcription of AcoACS1 in pineapple decreased after the ABRE was knocked out

**Figure 8:**
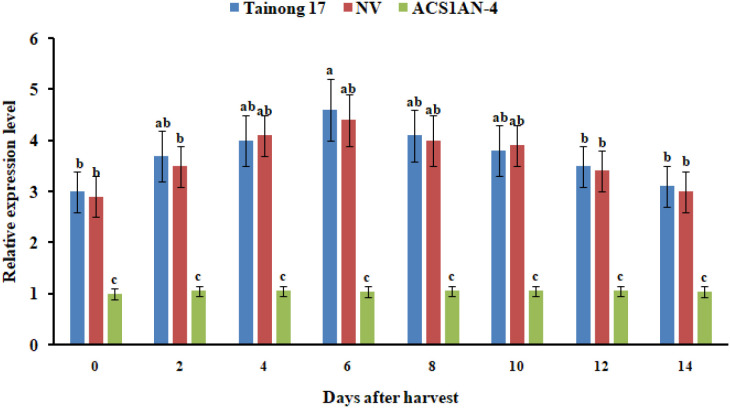
Relative expression levels of AcoACS1 in pineapple fruits. NV represents Tainong 17 fruits transformed with the empty vector. Pineapple fruits were collected at the 80% maturity stage and stored in a room at 25°C with 85% relative humidity. The measurements were performed with three replicates for each sample, including five to eight fruits per sample. Data are the means ± SE. Different letters indicate significant differences, which were analyzed according to Duncan’s test (*P* < 0.05).

To verify whether knocking out the ABRE can reduce enhancer function, the transcription levels of AcoACS1 in ACS1AN-4 fruits were measured. Results showed that the transcript levels of AcoACS1 in ACS1AN-4 were significantly lower than those in pineapple fruits transformed with the empty vector (NV) and Tainong 17 (**Figure 8**). These results indicated that after the sequence ACGT in the ABRE of the AcoACS1 enhancer was deleted, the transcript levels of AcoACS1 were significantly reduced, demonstrating that this ABRE is a core element of the enhancer. Its deletion can significantly impair the function of the enhancer.

### 3.6 Contents of main aroma compounds in the ACS1AN-4 fruit were similar to those in Tainong 17

To study whether knocking out the ABRE in AcoACS1 affects the edible quality of pineapple, fruits at 80% maturity were collected, and the contents of the main aroma compounds were measured. Results showed that the contents of main aroma compounds measured in ACS1AN-4 fruits were similar to those in Tainong 17 fruits (**Table 1**). For example, the content of benzyl acetate in the Tainong 17 fruit at 80% maturity was 21.86 ± 3.21 µg/kg. This value in the ACS1AN-4 fruit was 20.04 ± 3.46 µg/kg. However, the content of benzyl acetate in the ACS1–2 fruit at the same stage was 5.32 ± 0.65 µg/kg (**Table 1**). These results demonstrated that knocking out the ABRE reduced the enhancer function and ethylene content in pineapple fruits without destroying the function of AcoACS1. This allowed ACS1AN-4 fruits to maintain a certain amount of ethylene, which maintained the contents of main aromatic substances in the ACS1AN-4 pineapple fruit, thereby not affecting its edible quality.

**Table 1:** Contents of aroma substances in pineapple fruits (µg/kg).

Compound	Tainong 17	ACS1-2	ACS1AN-4
Benzyl acetate	21.86 ± 3.21 a	5.32 ± 0.65 b	20.04 ± 3.46 a
Methyl pentanoate	26.9 ± 3.53 a	3.67 ± 1.06 b	24.8 ± 2.64 a
Ethyl butyrate	46.64 ± 5.23 a	11.29 ± 2.35 b	45.92 ± 3.35 a
Diethyl succinate	685.96 ± 10.52 a	157.52 ± 12.63 b	671.65 ± 25.88 a
Z-4-Octenyl acetate	100.99 ± 6.33 a	32.36 ± 3.22 b	99.64 ± 6.20 a
Methyl valerate	380.35 ± 15.38 a	118.61 ± 7.36 b	375.55 ± 16.37 a
Methyl hexanoate	1,519.73 ± 73.90 a	267.02 ± 15.62 b	1,513.62 ± 55.03 a
Phenethyl acetate	1,182.63 ± 36.39 a	572.55 ± 26.65 b	1,180.11 ± 47.31 a
Phenyl acetate	27.02 ± 2.25 a	11.73 ± 2.04 b	25.92 ± 3.20 a
Ethyl phenylacetate	124.57 ± 8.09 a	35.72 ± 2.88 b	121.73 ± 9.89 a
Ethyl isobutyrate	365.93 ± 17.36 a	79.61 ± 5.32 b	359.03 ± 10.36 a
Ethyl crotonate	40.01 ± 3.62 a	28.62 ± 2.28 b	38.82 ± 5.20 a
Ethyl caproate	11.02 ± 2.06 a	5.92 ± 0.55 b	10.52 ± 1.03 a
Acetic acid phenyl ester	20.01 ± 3.35 a	8.06 ± 0.97 b	18.89 ± 1.35 a
Dimethyl phthalate	409.55 ± 15.32 a	128.93 ± 11.02 b	403.65 ± 13.26 a
10-Methylundecanoic acid methyl ester	50.06 ± 5.53 a	25.57 ± 3.96 b	46.27 ± 4.55 a
Methyl octanoate	27.71 ± 3.08 a	26.83 ± 3.27 a	27.14 ± 2.46 a
Ethyl 2-methylbutyrate	31.66 ± 3.39 a	21.84 ± 2.25 b	31.57 ± 2.17 a
3-Methylbutyl acetate	510.63 ± 21.50 a	213.72 ± 17.62 b	507.48 ± 17.73 a
3-Methylbutyl propanoate	134.06 ± 13.88 a	38.63 ± 3.61 b	133.52 ± 9.40 a
2,4-Dimethylpentyl acetate	31.08 ± 2.34 a	30.22 ± 2.99 a	29.36 ± 3.28 a
DL-3-Ethylhexanoic acid ethyl ester	13.05 ± 2.05 a	6.53 ± 0.93 b	11.03 ± 0.74 a
Eugenol	12.44 ± 1.87 a	11.89 ± 1.35 a	10.85 ± 0.66 a
Isobutyl isobutyrate	7.31 ± 1.06 a	3.26 ± 0.27 b	5.97 ± 0.53 a
Isoamyl formate	112.35 ± 12.85 a	106.92 ± 9.50 a	110.05 ± 7.20 a
Phenethyl acetate	33.52 ± 3.69 a	15.52 ± 1.04 b	31.16 ± 2.11 a
Cyclohexene oxide	103.11 ± 13.36 a	100.62 ± 6.40 a	100.07 ± 7.43 a
Dehydro-linalool	17.63 ± 2.63 a	16.62 ± 1.26 a	14.96 ± 1.03 a
1-Decene	22.03 ± 2.89 a	21.05 ± 1.34 a	17.93 ± 1.16 a
Citronellal	171.63 ± 11.06 a	170.09 ± 12.15 a	168.87 ± 9.40 a
alpha-Terpinene	222.26 ± 14.33 a	221.95 ± 18.76 a	213.52 ± 13.02 a
beta-Myrcene	163.36 ± 12.07 a	160.1 ± 13.36 a	156.66 ± 11.15 a
2,6-Dimethyl-2,4,6-octatriene	47.26 ± 4.73 a	45.51 ± 4.43 a	44.27 ± 4.47 a
2,4-Dimethylstyrene	7.55 ± 0.85 a	7.05 ± 1.00 a	7.05 ± 0.83 a
3,7-Dimethyl-1,3,6-octatriene	62.99 ± 4.75 a	60.07 ± 7.42 a	60.88 ± 5.52 a
Nerolidol	818.37 ± 22.70 a	817.76 ± 23.51 a	817.59 ± 15.30 a
alpha-Copaene	972.53 ± 26.35 a	971.93 ± 25.07 a	958.52 ± 19.94 a
alpha-Terpinene	53.36 ± 6.63 a	50.08 ± 3.70 a	51.16 ± 2.55 a
alpha-Pinene	20.05 ± 2.16 a	19.27 ± 2.53 a	19.36 ± 1.39 a

The measurements were performed with three replicates for each sample, with five to eight fruits per replicate. Data are the means (*n* = 3) ± SE. Different letters in the same row indicate signiﬁcant differences determined by Duncan’s test (*P* < 0.05).

### 3.7 Knocking out the ABRE in the AcoACS1 enhancer extended the shelf-life of pineapple fruits

**Figure 9:**
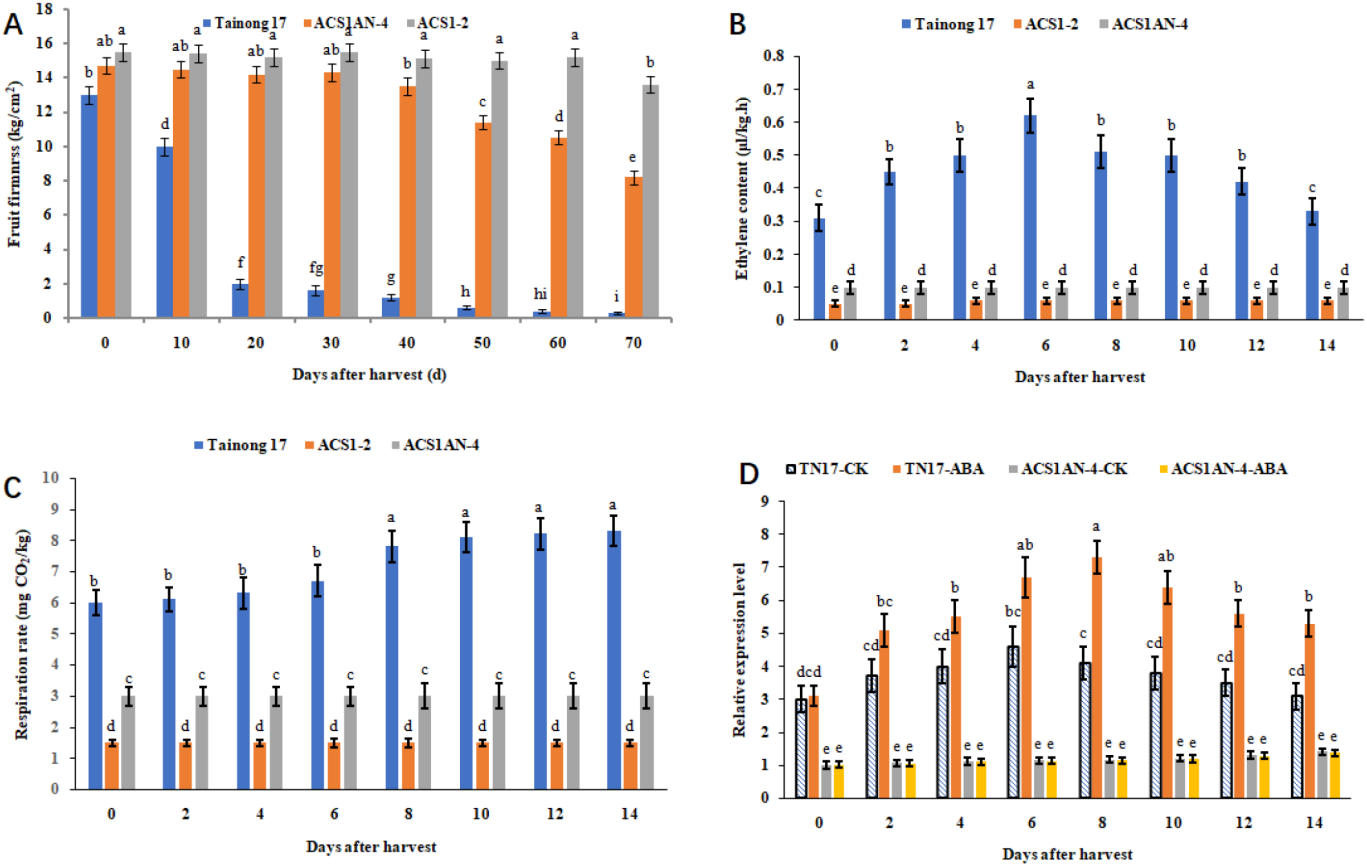
Knocking out the ABRE in AcoACS1 extends the shelf life of pineapple. **(A)** Fruit firmness of different pineapple lines. **(B)** Ethylene content. **(C)** Respiration rate. **(D)** Relative expression level of AcoACS1. CK represents fruit sprayed with 50 mL of water. ABA represents fruit sprayed with 50 mL of 0.1 mol/L ABA. TN17 represents Tainong 17. Pineapple fruits were harvested at the 80% maturity stage and stored at 25 °C with 85% relative humidity. Three biological replicates were performed for each treatment, each consisting of five to eight fruits. Data are presented as means ± SE. Different letters indicate significant differences according to Duncan’s test (*P* < 0.05).

To investigate the effect of knocking out the ABRE in the AcoACS1 enhancer on pineapple softening, fruit firmness was measured using a TMS-Pro food texture analyzer. The results showed that the firmness of the Tainong 17 fruit, harvested at 80% maturity, began to decline immediately after harvest and continued to decrease when stored at 25°C with 85% relative humidity. By the 20th day after harvest, the Tainong 17 fruit had completely rotted. In contrast, the firmness of the ACS1AN-4 fruit showed no significant change within 40 days after harvest (**Figure 9A**). After 40 days, the firmness decreased but remained significantly higher than that of Tainong 17. Similarly, the firmness of the ACS1–2 fruit showed no significant change within 60 days (**Figure 9A**), after which it began to decrease.

Ethylene content and respiration rate play decisive roles in fruit ripening and softening (Dos Santos et al., 2019). To investigate whether knocking out the ABRE affects ethylene content and respiration rate in pineapple fruit, these parameters were measured in fruits harvested at 80% maturity in this research. The results showed that ethylene content in the ACS1AN-4 fruit was significantly lower than that in the Tainong 17 fruit but higher than that in the ACS1–2 fruit (**Figure 9B**). A similar trend was observed for the respiration rate (**Figure 9C**). These findings indicated that knocking out the ABRE in the AcoACS1 enhancer reduced its activity, thereby decreasing ethylene synthesis. However, AcoACS1 remained functional, though its transcription rate is lower than that in Tainong 17.

### 3.8 ABA regulates pineapple fruit softening via AcoACS1

Pineapple is a non-climacteric fruit, and ABA treatment can promote its softening. Knocking out the ABRE in AcoACS1 may block this promotional effect. To test this hypothesis, AcoACS1 expression levels were measured in pineapple fruits sprayed with 50 mL of 0.1 mol/L ABA at different stages. The results showed that after ABA treatment, AcoACS1 expression in Tainong 17 fruit increased significantly, whereas no significant difference was observed in ACS1AN-4 fruits sprayed with ABA compared to those sprayed with water (**Figure 9D**). Tainong 17 fruits sprayed with ABA softened faster than those treated with water. Moreover, Tainong 17 fruits treated with ABA softened faster than ACS1AN-4 fruits treated with ABA. The firmness of the ACS1AN-4 fruit treated with ABA was similar to that of fruit treated with water. These results demonstrate that ABA promotes AcoACS1 expression and accelerates fruit softening. Following the knockout of the ABRE sequence, AcoACS1 expression in the fruit was unaffected by ABA treatment. This indicates that ABA promotes pineapple softening via the ABRE in the AcoACS1 enhancer. ABA regulates pineapple softening through AcoACS1.

### 3.9 Hydrolase activities in ACS1AN-4 fruits were lower than those in Tainong 17

The composition and structure of flesh cell walls are the main factors in maintaining fruit firmness (Tucker et al., 2017). The degradation of cell walls by hydrolases is the primary cause of fruit softening. To investigate the mechanism by which the ABRE regulates pineapple softening, hydrolase activities in pineapple flesh were measured. Results showed that the activities of PG, PME, GAL, XYL, and CX in the ACS1AN-4 fruit were significantly lower than those in Tainong 17 (**Table 2**). However, the activities of these hydrolases in the ACS1AN-4 fruit were higher than those in ACS1-2 (**Table 2**). These results indicated that the ABRE promotes the activities of endogenous hydrolases and regulates the fruit softening process. Knocking out the ABRE in the AcoACS1 enhancer inhibits endogenous hydrolase activities, thereby delaying pineapple softening.

**Table 2:** Hydrolase activities of endogenous hydrolases in pineapple fruit at different stages ((µg gal acid)/(mg protein h)).

Period	PG	PME	GAL	XYL	CX
TN17-I	11.5 ± 0.3 d	0.34 ± 0.03 a	0.12 ± 0.02 b	1.25 ± 0.03 c	1.29 ± 0.03 b
TN17-II	12.7 ± 0.4 c	0.36 ± 0.03 a	0.17 ± 0.03 ab	1.42 ± 0.03 bc	1.42 ± 0.04 a
TN17-III	14.9 ± 0.5 a	0.37 ± 0.04 a	0.22 ± 0.04 a	1.55 ± 0.04 a	1.02 ± 0.02 c
TN17-IV	14.2 ± 0.4 ab	0.38 ± 0.04 a	0.21 ± 0.04 ab	1.47 ± 0.03 b	1.42 ± 0.04 a
TN17-V	13.5 ± 0.3 b	0.36 ± 0.04 a	0.18 ± 0.02 ab	1.18 ± 0.03 d	1.25 ± 0.03 b
AN-I	3.2 ± 0.1 e	0.13± 0.01 b	0.04 ± 0.01 c	0.60 ± 0.02 e	0.71 ± 0.02 d
AN-II	3.5 ± 0.1 e	0.13 ± 0.01 b	0.04 ± 0.01 c	0.60 ± 0.02 e	0.70 ± 0.02 d
AN-III	3.3 ± 0.2 e	0.13 ± 0.01 b	0.04 ± 0.01 c	0.60 ± 0.02 e	0.72 ± 0.02 d
AN-IV	3.4 ± 0.2 e	0.13 ± 0.02 b	0.04 ± 0.01 c	0.61 ± 0.02 e	0.71 ± 0.03 d
AN-V	3.5 ± 0.2 e	0.13 ± 0.02 b	0.04 ± 0.02 c	0.61 ± 0.03 e	0.71 ± 0.03 d
ACS-I	1.2 ± 0.1 f	0.05 ± 0.01 c	0.02 ± 0.01 c	0.20 ± 0.02 f	0.21 ± 0.02 e
ACS-II	1.5 ± 0.1 f	0.05 ± 0.01 c	0.02 ± 0.01 c	0.20 ± 0.02 f	0.21 ± 0.02 e
ACS-III	1.3 ± 0.2 f	0.05 ± 0.01 c	0.02 ± 0.01 c	0.20 ± 0.02 f	0.22 ± 0.02 e
ACS-IV	1.4 ± 0.2 f	0.05 ± 0.02 c	0.02 ± 0.01 c	0.20 ± 0.02 f	0.21 ± 0.03 e
ACS-V	1.5 ± 0.2 f	0.05 ± 0.02 c	0.02 ± 0.02 c	0.20 ± 0.03 f	0.22 ± 0.03 e

I represents the stage where the green peel area was more than 95% of the total surface area. II represents the stage where the green peel area accounted for 80%–90% of the total surface area. III represents the stage where the yellow peel area accounted for 20%–40% of the total surface area. IV represents the stage where the yellow peel area accounted for 60%–90% of the total surface area. V represents the stage where the entire peel was yellow. AN represents ACS1AN-4. TN17 represents Tainong 17. ACS represents the AcoACS1 mutant ACS1-2. Measurements were performed with three replicates for each sample, each consisting of five to eight fruits. Data are presented as means ± SE (*n* = 3). Different letters in the same column indicate significant differences according to Duncan’s test (*P* < 0.05).

### 3.10 The mechanism by which the ABRE in AcoACS1 regulates pineapple softening

**Figure 10:**
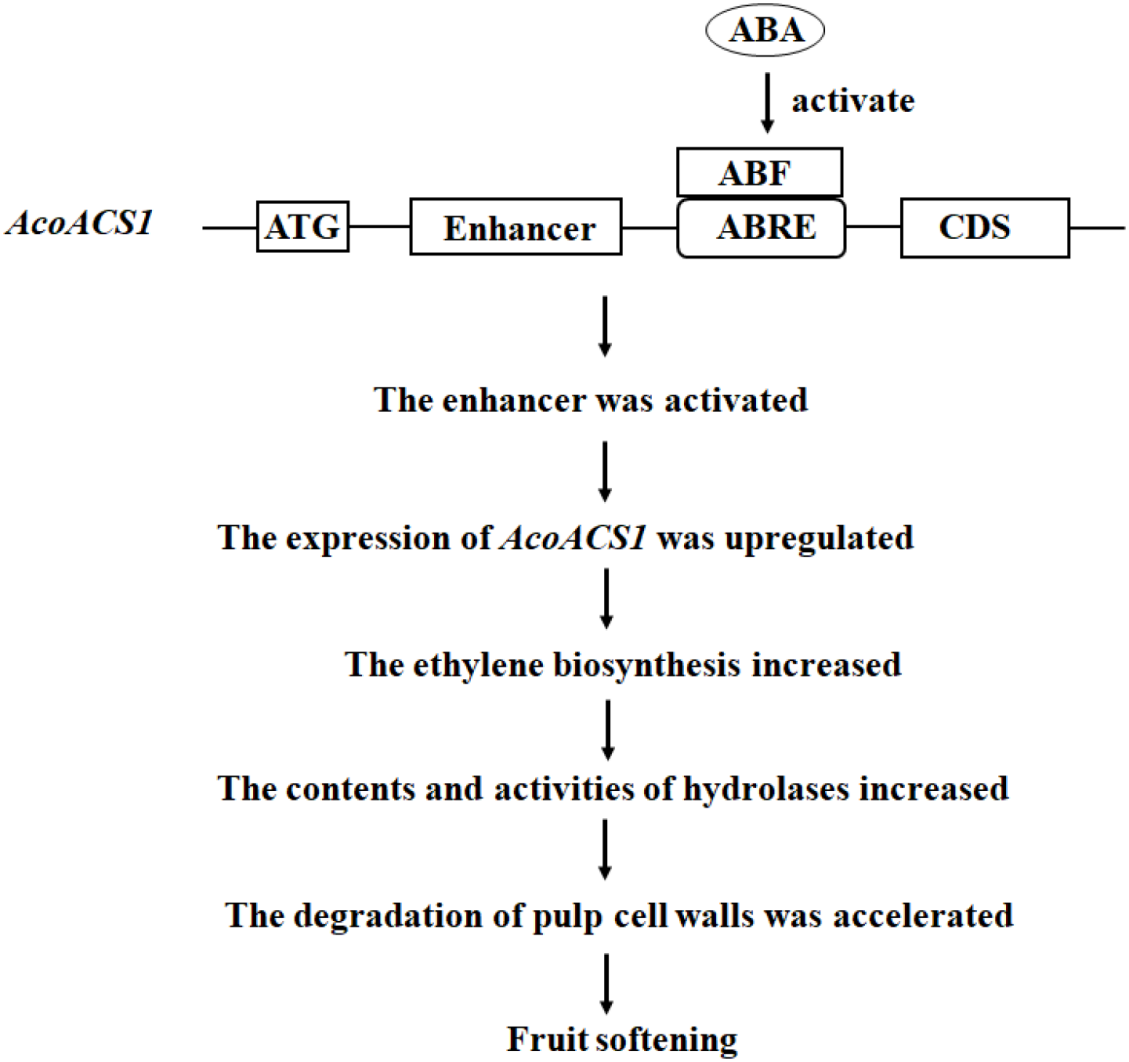
The proposed mechanism by which ABA regulates pineapple softening. ABF represents the ABRE-binding factor.

Based on the results described above, a mechanism by which ABA regulates pineapple softening through the ABRE in AcoACS1 was proposed (**Figure 10**). First, ABA activates ABRE-binding factors (ABFs) (Nakashima and Yamaguchi-Shinozaki, 2013). ABFs then bind to the ABRE, enhancing AcoACS1 expression, which subsequently increases ethylene synthesis. Increased ethylene levels in pineapple fruit stimulate the respiration rate (Dos Santos et al., 2019). This accelerates the production of ATP and NADPH, providing energy for the synthesis and activation of hydrolases. Consequently, hydrolase levels and activities increase, accelerating the degradation of flesh cell walls and leading to fruit softening.

## 4 Discussion

Shelf life is an important phenotype for evaluating pineapple cultivars. A lot of pineapples are discarded because of softening in China each year. Creating germplasm with an extended shelf life is important for the Chinese pineapple industry. Pineapple is a non-climacteric fruit, of which its ripening and softening are regulated by ABA (Fan et al., 2022). However, research demonstrated that ethylene also participates in this process (Zhang et al., 2016a; Karagiannis et al., 2018; Liu et al., 2015). Since ethylene biosynthesis in plants contains fewer steps than that of ABA (Dong et al., 2015; Larsen, 2015), we hypothesized that regulating key genes in ethylene biosynthesis is a more feasible strategy for creating pineapple germplasm with a long shelf life. In this study, the pineapple ethylene synthase gene AcoACS1 was mutated, and the mutant line ACS1–2 was generated. During ripening, the ethylene content in the ACS1–2 fruit was significantly lower than that in the Tainong 17 fruit. The onset of softening in the ACS1–2 fruit was significantly later than that of Tainong 17. However, the ACS1–2 fruit lacked the characteristic aroma of the Tainong 17 fruit, and its commercial value was inferior to that of Tainong 17. Analysis revealed that the levels of major aroma substances in the ACS1–2 fruit were far lower than those in the Tainong 17 fruit. For instance, the benzyl acetate content in the Tainong 17 fruit at 80% maturity was 21.86 ± 3.21 µg/kg, whereas in the ACS1–2 fruit at the same stage, it was only 5.32 ± 0.65 µg/kg. This severely compromised the fruit’s aromatic quality. Although the ACS1–2 fruit exhibited a longer shelf life than Tainong 17, the reduction in the levels of major aroma substances limits its commercial potential.

Ethylene plays a crucial role in the synthesis of aromatic substances in plants (Tipu and Sherif, 2024). The ethylene content in the ACS1–2 fruit was significantly lower than that in Tainong 17, which might explain why the levels of major aroma compounds in the ACS1–2 fruit were lower than those in Tainong 17. If a pineapple germplasm were created with lower ethylene content than Tainong 17, but with ethylene levels still sufficient to initiate the biosynthesis of aroma compounds, the resulting fruit would have a long shelf life without compromising its edible quality.

There is an enhancer sequence containing an ABRE site in the genomic sequence of AcoACS1. ABA can activate ABF (Nakashima and Yamaguchi-Shinozaki, 2013), which facilitates the binding of ABF to ABRE, promoting the transcriptional expression of the gene harboring this element (Fujita et al., 2013). We hypothesized that the ABRE site plays a crucial role in pineapple fruit ripening and softening by regulating the expression of AcoACS1. Knocking out this ABRE site would disrupt ABA regulation of ripening and softening while allowing AcoACS1 expression to persist. This strategy was expected to achieve two outcomes: firstly, the onset of pineapple softening was delayed, and the shelf life was extended; secondly, sufficient ethylene was synthesized to activate the biosynthesis of aroma compounds. Our experiments confirmed this hypothesis. The ethylene content in the fruit of the AcoACS1 ABRE knockout mutant (ACS1AN-4) was significantly lower than that in Tainong 17, but higher than that in ACS1-2. The onset of softening in the ACS1AN-4 fruit occurred much later than that in Tainong 17, but earlier than in ACS1-2. No significant difference was found in the levels of major aroma substances between Tainong 17 and ACS1AN-4 fruits. For instance, the 3-methylbutyl propanoate content in the Tainong 17 fruit was 134.06 ± 13.88 µg/kg, while that in the ACS1AN-4 fruit was 133.52 ± 9.40 µg/kg. Similarly, the ethyl 2-methylbutyrate content in the Tainong 17 fruit was 31.66 ± 3.39 µg/kg, compared to 31.57 ± 2.17 µg/kg in the ACS1AN-4 fruit.

The composition and structure of cell walls are the main factors in maintaining fruit firmness (Tucker et al., 2017). The main components of cell walls are cellulose, pectin, and lignin (Tucker et al., 2017). Enhanced activity of endogenous hydrolases plays a major role in fruit softening (Sozzi et al., 2001; Han et al., 2016; Wang et al., 2020). Endogenous hydrolases in pineapple fruit mainly include PG, PME, GAL, XYL, and CX (Ye et al., 2014). Their activities are regulated by ethylene (Li et al., 2020; Wang et al., 2020; Han et al., 2016). Increased ethylene content enhances cell respiration and the activity of endogenous hydrolases, leading to the degradation of flesh cell walls (Chen et al., 2018; Zhang et al., 2018) and subsequent fruit softening (Fabi and do Prado, 2019; Brizzolara et al., 2020). The activities of PG, PME, GAL, XYL, and CX in ACS1AN-4 fruits were significantly lower than those in Tainong 17, but were higher than those in ACS1–2 fruits. PG activity in Tainong 17 fruits at the stage when the yellow area accounted for 20%–40% of the total surface area was 14.9 ± 0.5 µg gal acid/(mg protein·h). The corresponding values in ACS1AN-4 and ACS1–2 fruits at the same stage were 3.3 ± 0.2 and 1.3 ± 0.2 µg gal acid/(mg protein·h), respectively. This indicated that knocking out the ABRE inhibited the activities of endogenous hydrolases. Pineapple fruit softening can be regulated through AcoACS1 and ethylene. The initiation of pineapple softening can be delayed by knocking out the ABRE in AcoACS1.

There is a crosstalk between the biosynthesis of ethylene and ABA in plants (Weng et al., 2015). The AcoACS1 genomic sequence contains an ABRE element, which mediates regulation by ABA. Knockout of the ABRE element within AcoACS1 provided an opportunity to investigate whether ethylene influences ABA content in pineapple fruit. ABA content in ACS1AN-4, ACS1-2, and Tainong 17 fruits at different stages was measured in this study. Results revealed that ABA levels in ACS1AN-4 and ACS1–2 fruits were significantly lower than those in Tainong 17 across different stages (**Supplementary Table S1**). This indicates that ABA biosynthesis in pineapple fruit is positively regulated by ethylene.

Currently, reported methods for enhancer editing include saturation mutagenesis and CRISPR/Cas9 RNP (Canver et al., 2015; Fu et al., 2022; Zeng et al., 2025). The mechanism underlying saturation mutagenesis involves designing a vast library of sgRNAs that tile across a target enhancer region, ensuring coverage of every nucleotide. This extensive library is introduced into cells co-expressing the Cas9 nuclease. Upon Cas9-mediated DNA cleavage, the cell’s endogenous repair machinery, primarily non-homologous end joining (NHEJ), is engaged. As NHEJ is often error-prone, it results in random insertions or deletions. Consequently, this process generates a diverse array of mutations across every base within the target region. The objective of saturation mutagenesis is to identify critical regulatory sites within an enhancer. This study aims to characterize the function of the ABRE located within the AcoACS1 enhancer. This ABRE motif comprises only five base pairs. A PAM is situated two nucleotides immediately downstream of this ABRE, enabling targeted mutagenesis of the locus. Thus, rather than screening for unknown sites, our approach focuses on mutating a predefined locus. This objective can be achieved using the CRISPR/Cas9 system.

The mechanism of CRISPR/Cas9 RNP delivery was as follows. *In vitro*, purified Cas9 protein is complexed with synthetic sgRNA to form an active RNP complex. This pre-assembled complex possesses immediate DNA-cleavage capability. The RNP is delivered directly into the cell nucleus via physical methods, such as electroporation. Upon entering the nucleus, the RNP complex rapidly binds to the target DNA sequence, where the Cas9 nuclease induces a double-strand break. The cell’s endogenous repair mechanisms, predominantly NHEJ, then repair the break, often resulting in insertions or deletions that disrupt gene function. Unlike DNA-based vectors, RNPs consist of exogenous protein and RNA molecules. Intracellular proteases and nucleases rapidly recognize and degrade these components. Consequently, the intracellular half-life of RNPs is short, minimizing the risk of persistent off-target activity following the editing event. Although the CRISPR/Cas9 RNP system offers enhanced safety profiles, the presence of a rigid cell wall in plants poses a significant barrier to the direct nuclear delivery of RNP complexes. With the development of pineapple protoplast regeneration techniques, CRISPR/Cas9 RNP may be applied in pineapple genome editing.

## Data availability statement

Publicly available datasets were analyzed in this study. This data can be found here: https://www.ncbi.nlm.nih.gov/nuccore/XM_020229961.1/ and https://www.ncbi.nlm.nih.gov/nuccore/NC_033623.1.

## Author contributions

SC: Validation, Project administration, Conceptualization, Funding acquisition, Writing – review & editing, Investigation, Writing – original draft, Supervision. HS: Writing – review & editing, Validation, Investigation, Formal analysis, Writing – original draft, Methodology, Data curation. AL: Writing – review & editing, Investigation. YW: Writing – review & editing, Investigation. JH: Validation, Writing – review & editing. QW: Validation, Writing – review & editing. RZ: Validation, Writing – review & editing.
