# Supplementary material for: Developing pineapples with an extended shelf life through deletion of the abscisic-acid-responsive element in the enhancer sequence of the 1-aminocyclopropane-1-carboxylate synthase gene AcoACS1
Source: Front Plant Sci. 2026 Feb 20;17:1769495. doi: 10.3389/fpls.2026.1769495 (PMC12963278; doi:10.3389/fpls.2026.1769495)
Supplement: Supplementary file 1 [file DataSheet1.docx]

Supplementary file


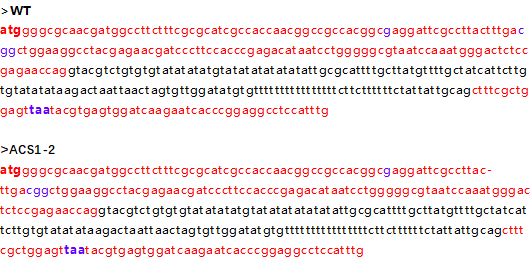


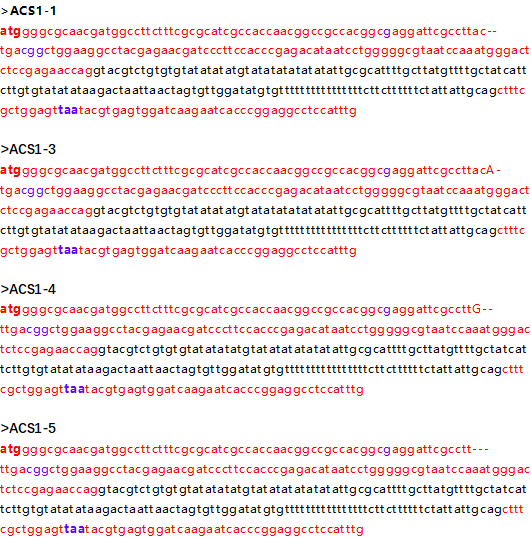


Figure S1. Target sequences of the AcoACS1 gene in pineapple lines.
The wild-type (WT) control was Tainong 17. The Protospacer Adjacent Motif (PAM) and the first nucleotide of the target sequences are highlighted in blue. Putative premature stop codons are indicated in bold blue. Deleted bases are represented by red dashes. Exons and introns are shown in red and black letters, respectively.


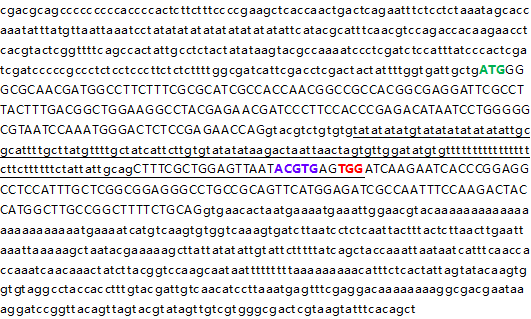


Figure S2. Partial genomic sequence of AcoACS1. The enhancer sequence is underlined. The ABRE sequence is shown in blue capital bold letters. The PAM site for constructing the ABRE-knockout line is shown in capital red letters. The start codon was shown in blue capital letters. The introns are shown in lowercase letters. The exons are shown in capital letters.

Table S1. ABA content in pineapple fruit (μg/g FW)

|  | 3 MAF | 4 MAF | 5 MAF | 6 MAF |
| --- | --- | --- | --- | --- |
| ACS1AN-4 | 6.0±0.5 b | 7.8±0.7 b | 12.6±0.8 b | 14.7±0.8 b |
| ACS1-2 | 4.2±0.7 c | 5.3±1.0 c | 9.4±1.1 c | 10.0±1.5 c |
| Tainong 17 | 16.1±0.9 a | 18.2±1.1 a | 33.5±1.2 a | 50.6±1.3 a |

Note: MAF refers to months after flower forcing. The measurements were conducted with three replicates for each sample, including 5-8 fruits per sample. Data are the means (n=3) ± SE. Different letters in the same row indicate signiﬁcant differences according to Duncan´s test (p< 0.05).
